# Cutaneous adnexal tumors: the diagnostic role of dermatoscopic examination

**DOI:** 10.3906/sag-2007-86

**Published:** 2021-04-30

**Authors:** Ömer Faruk ELMAS, Abdullah DEMİRBAŞ*, Ülker GÜL, Mustafa ATASOY

**Affiliations:** 1 Department of Dermatology and Venereology, Faculty of Medicine, Ahi Evran University, Kırşehir Turkey; 2 Department of Dermatology and Venereology, Konya Numune State Hospital, Konya Turkey; 3 Department of Dermatology and Venereology, Dışkapı Research and Training Hospital, Ankara Turkey; 4 Department of Dermatology and Veneorology, Health Science University, Kayseri City Hospital, Kayseri Turkey

We have read with great interest the original article by Aslan Kayıran et al., which was recently published in the Turkish Journal of Medical Sciences [1]. In their retrospective study, the authors investigated the clinical and histopathological characteristics of the patients with cutaneous adnexal tumors. They compared the clinical preliminary diagnoses and the final histopathological diagnoses of the cases to reveal the success of the clinicians in diagnosing cutaneous adnexal tumors. The results showed that clinical prediagnoses and histopathological diagnoses were compatible in 45% of the 65 patients, while they were contradictory in 51%. The authors concluded that cutaneous adnexal tumors should be included in the differential diagnosis list, particularly in the presence of cutaneous papulonodular lesions without specific features and that a histopathological examination must be done in suspicious cases to reach a definitive diagnosis [1]. Here, we would like to shortly discuss the contribution of dermatoscopic examination to diagnostic accuracy in cutaneous adnexal tumors.

Recently, dermatoscopy has become an essential tool in dermatology practice, and dermatoscopic characteristics of many neoplastic and nonneoplastic skin conditions have been well identified. Current studies showed that different types of cutaneous adnexal tumors may have peculiar dermatoscopic features. For example, sebaceous nevus dermatoscopically shows yellowish clods arranged in cobblestone pattern corresponding to conglomerations of dermal hyperplastic sebaceous glands in the histopathology [2]. Sebaceous hyperplasia exhibits central white to yellowish clods along with a surrounding crown of vessels histopathologically corresponding to hyperplastic sebaceous glands and dermal telangiectatic vessels [3] (Figure a). Trichofolliculoma may have a dermatoscopic pattern reminiscent of “rosary bead with tassel”-like appearance [4]. Apocrine hidrocystoma presents with central skin-colored to blue translucent structureless area histopathologically corresponding to a large cystic space located within the dermis [3] (Figure b). Eccrine hidrocystoma usually displays multiple gray to bluish structureless areas [3] (Figure c). Poroma typically exhibits complex irregular vessels with bulbous endings and white interlacing lines [3] (Figure d). Irregularly distributed white to yellow structureless areas histopathologically corresponding to calcification, white streaks, reddish structureless areas, and linear irregular vessels represent the most common dermatoscopic findings of pilomatrixoma [3]. It is noteworthy to mention that there is apparently no comprehensive study investigating the exact histopathological counterparts of dermoscopic findings observed in cutaneous adnexal tumors. Most of the reports on the subject are based on expert opinions or case studies focused on a peculiar tumor. The absence of distinctive dermatoscopic findings in a considerable part of cutaneous adnexal tumors may explain the paucity of extensive studies on the subject. Prospective studies with large sample sizes focused on the dermatoscopic and histopathological correlation may open a new horizon in the diagnosis of cutaneous adnexal tumors.

**Figure:**
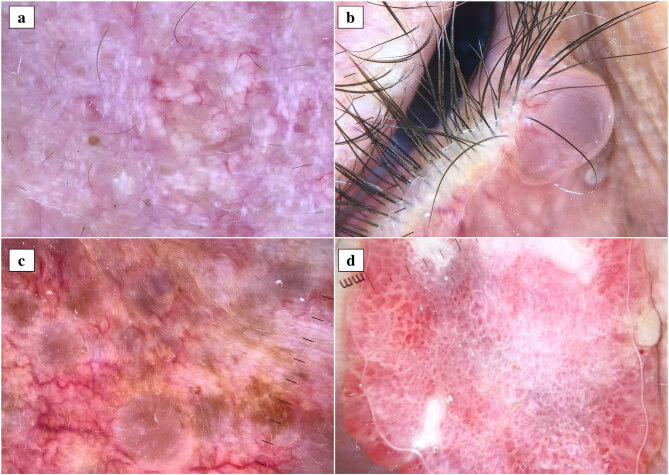
Dermatoscopic images of different types of cutaneous adnexal tumors. Sebaceous hyperplasia exhibits central white to yellowish clods along with a surrounding crown of vessels (a). Apocrine hidrocystoma shows skin-colored to bluish translucent structureless area with irregular linear vessels (b). Eccrine hidrocystoma displays multiple gray to bluish structureless areas (c). Poroma shows complex irregular vessels with bulbous endings and white interlacing lines (d).

The diagnosis of cutaneous adnexal tumors is usually not straightforward on the clinical background. Even with histopathological examination, some cases may remain undiagnosed. In this regard, a dermatoscopic examination may provide useful clues for the differential diagnosis. However, by relying only on dermatoscopic examination, malignant adnexal tumors, such as porocarcinoma and sebaceous carcinoma, can be confused with benign ones. Besides, a tumor that was previously benign can become malignant later. Some of the cutaneous adnexal tumors may dermatoscopically mimic different types of epithelial tumors, particularly basal cell carcinoma. In this context, dermatoscopic findings should always be interpreted in the setting of clinical features, and histopathological examination should always be performed in suspicious cases. Strong cooperation between dermatologists and pathologists may increase the rate of precise histopathological diagnosis, particularly in hard-to-diagnose cases.
